# Distinct profiles of LRRK2 activation and Rab GTPase phosphorylation in clinical samples from different PD cohorts

**DOI:** 10.1038/s41531-022-00336-5

**Published:** 2022-06-08

**Authors:** Lilian Petropoulou-Vathi, Athina Simitsi, Politymi-Eleni Valkimadi, Maria Kedariti, Lampros Dimitrakopoulos, Christos Koros, Dimitra Papadimitriou, Alexandros Papadimitriou, Leonidas Stefanis, Roy N. Alcalay, Hardy J. Rideout

**Affiliations:** 1 Center for Clinical, Experimental Surgery, and Translational Research, Biomedical Research Foundation of the Academy of Athens, Athens, Greece; 2 Department of Neurology, University of Athens Medical School, Athens, Greece; 3 Department of Neurology, Henry Dunant Hospital Centre, Athens, Greece; 4 Department of Neurology, Columbia University, York City, NY USA

**Keywords:** Diseases of the nervous system, Proteins

## Abstract

Despite several advances in the field, pharmacodynamic outcome measures reflective of LRRK2 kinase activity in clinical biofluids remain urgently needed. A variety of targets and approaches have been utilized including assessments of LRRK2 itself (levels, phosphorylation), or its substrates (e.g. Rab10 or other Rab GTPases). We have previously shown that intrinsic kinase activity of LRRK2 isolated from PBMCs of G2019S carriers is elevated, irrespective of disease status. In the present study we find that phosphorylation of Rab10 is also elevated in G2019S carriers, but only those with PD. Additionally, phosphorylation of this substrate is also elevated in two separate idiopathic PD cohorts, but not in carriers of the A53T mutation in α-synuclein. In contrast, Rab29 phosphorylation was specifically reduced in urinary exosomes from A53T and idiopathic PD patients. Taken together, our findings highlight the need for the assessment of multiple complimentary targets for a more comprehensive picture of the disease.

## Introduction

The kinase activity of leucine-rich repeat kinase 2 (LRRK2) has emerged as the primary target for both therapeutic development and biomarker design in Parkinson’s disease (PD). Both cellular and animal models have demonstrated that neurodegeneration induced by mutant forms of LRRK2 is kinase activity-dependent (e.g. ref. ^1^). Similarly, early biomarker studies, assessing LRRK2 auto-phosphorylation (pS1292-LRRK2) in urinary exosomes, have indicated that LRRK2 activity may be elevated during the clinical progression of LRRK2-associated, as well as idiopathic, PD (iPD)^2,3^. In addition to auto-phosphorylation of LRRK2 and its intrinsic kinase activity, which are measures of distinct aspects of its kinase function, other readouts of the activation state of LRRK2 include phosphorylation of endogenous substrates, such as members of the Rab family of small GTPases^4^. Extending these findings, the group of Greenamyre and colleagues^5^ applied a novel proximity ligation assay to detect pS1292-LRRK2 and pT73-Rab10 in ventral midbrain of post mortem tissue from iPD patients. In subsequent studies, we have reported that the in vitro kinase activity of purified LRRK2 from peripheral blood mononuclear cells (PBMCs) of G1029S-LRRK2 mutation carriers is elevated^6^. Alternatively, while phosphorylation at the S935 site within the N-terminal region of LRRK2 is a robust measure of target engagement (e.g. refs. ^7,8^), it does not completely track with kinase activity of LRRK2^9^.

Rab10 phosphorylation has been assessed in various PD cohorts, however the findings have been mixed. In a small proof-of-concept study, Rab10 phosphorylation (T73) in neutrophils was not significantly changed in carriers of the G2019S LRRK2 mutation^10^; and in PBMCs from iPD patients, pT73-Rab10 was not changed compared to healthy controls, despite being correlated with levels of certain cytokines^11^. Interestingly, in neutrophils from carriers of the less common R1441G mutation in LRRK2, a significant increase in pT73-Rab10 is detected in comparison to healthy controls, irrespective of disease status^12^. This is consistent with findings reported in transgenic mice expressing R1441C or G2019S, where a robust increase in Rab10 phosphorylation was observed in R14411C but not G2019S mice^13^. Most recently, using a proprietary antibody raised against pT73-Rab10, Wang and colleagues reported that phosphorylated Rab10 levels, normalized to total LRRK2, are increased in PBMCs of affected carriers of the G2019S mutation^8^ and are reduced in cells treated ex vivo with LRRK2 kinase inhibitors.

In the present study, we find increased Rab10 phosphorylation in iPD patients and affected G2019S-*LRRK2* carriers only, whereas levels in healthy carriers and A53T-*SNCA* PD patients are not changed from healthy controls. Additionally, phosphorylation of Rab29 is decreased in urinary exosomes from *SNCA* mutation carriers and iPD patients. Thus, it appears that alterations in peripheral LRRK2-dependent Rab phosphorylation are not uniformly linked to either disease status or genotype.

## Results

### Phosphorylation of Rab10 is increased in PBMCs of iPD and G2019S-LRRK2 carriers with PD

**Fig. 1:**
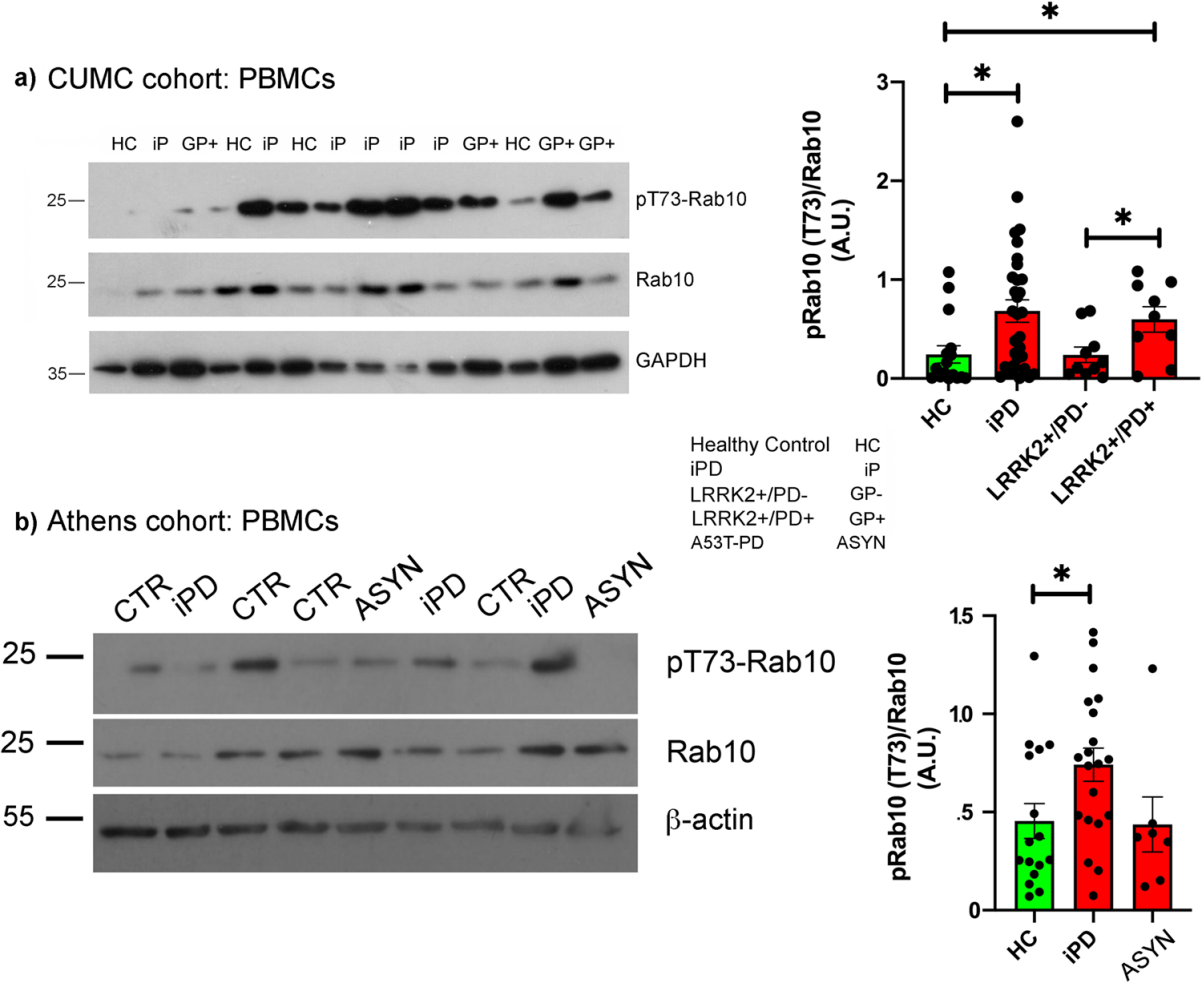
Phosphorylation of Rab10 is elevated in PBMCs from idiopathic PD and affected carriers of the G2019S mutation in LRRK2. Extracts of PBMCs from the indicated subject groups were separated by SDS-PAGE and probed for phosphorylated (T73) and total Rab10, as well as GAPDH. **a** Representative western immunoblots showing pT73-Rab10 and total Rab10 from PBMCs of the CUMC cohort; with the quantification of the band intensity shown to the right. ANOVA was performed to identify significant differences between groups, with Tukey’s post-foc tests. *p < 0.05. **b** PBMC extracts were separated by SDS-PAGE. A representative western immunoblot probed for pT73-Rab10 and total Rab10 from PBMCs of the Athens cohort; with the band intensities quantified and presented in the panel to the right. For normalization, the band intensity of pT73-Rab10 was then normalized to total Rab10. Error bars in all data plots represent SEM. ANOVA was performed to identify significant differences between groups, with Tukey’s post-foc tests. *p < 0.05.

By Western immunoblotting, we assessed levels of pT73-Rab10 and pT71-Rab29 in PBMC extracts from each group. In Fig. 1a, a representative immunoblot showing levels of phosphorylated (T73) and total Rab10 from each group from the Columbia cohort samples is shown, along with quantification of the bands. In Fig. 1b, we show similar representative blots from the Athens cohort and their accompanying quantification. When normalized to total Rab10 levels, we found a significant increase in pT73-Rab10 in idiopathic PD (iPD) from both the Columbia cohort, as well as the Athens cohort, compared to healthy control subjects (Fig. 1a, b). We treated a parallel set of PBMCs with the LRRK2 inhibitor MLi2, and found a significant reduction in pRab10 levels compared to untreated cells (Supplementary Fig. 2a), indicating that in these cells Rab10 phosphorylation is largely mediated by LRRK2 kinase activity. Like LRRK2 expression^6^, expression of Rab10 (total) was not significantly altered in either iPD cohort. As an alternative approach, similar to what has been reported by Wang and colleagues^8^, we normalized pRab10 to total LRRK2 expression; however, while the same trends were retained (i.e. G2019S+ PD+ and iPD levels were slightly increased) the differences were not statistically significant (Supplementary Fig. 1c).

**Fig. 2:**
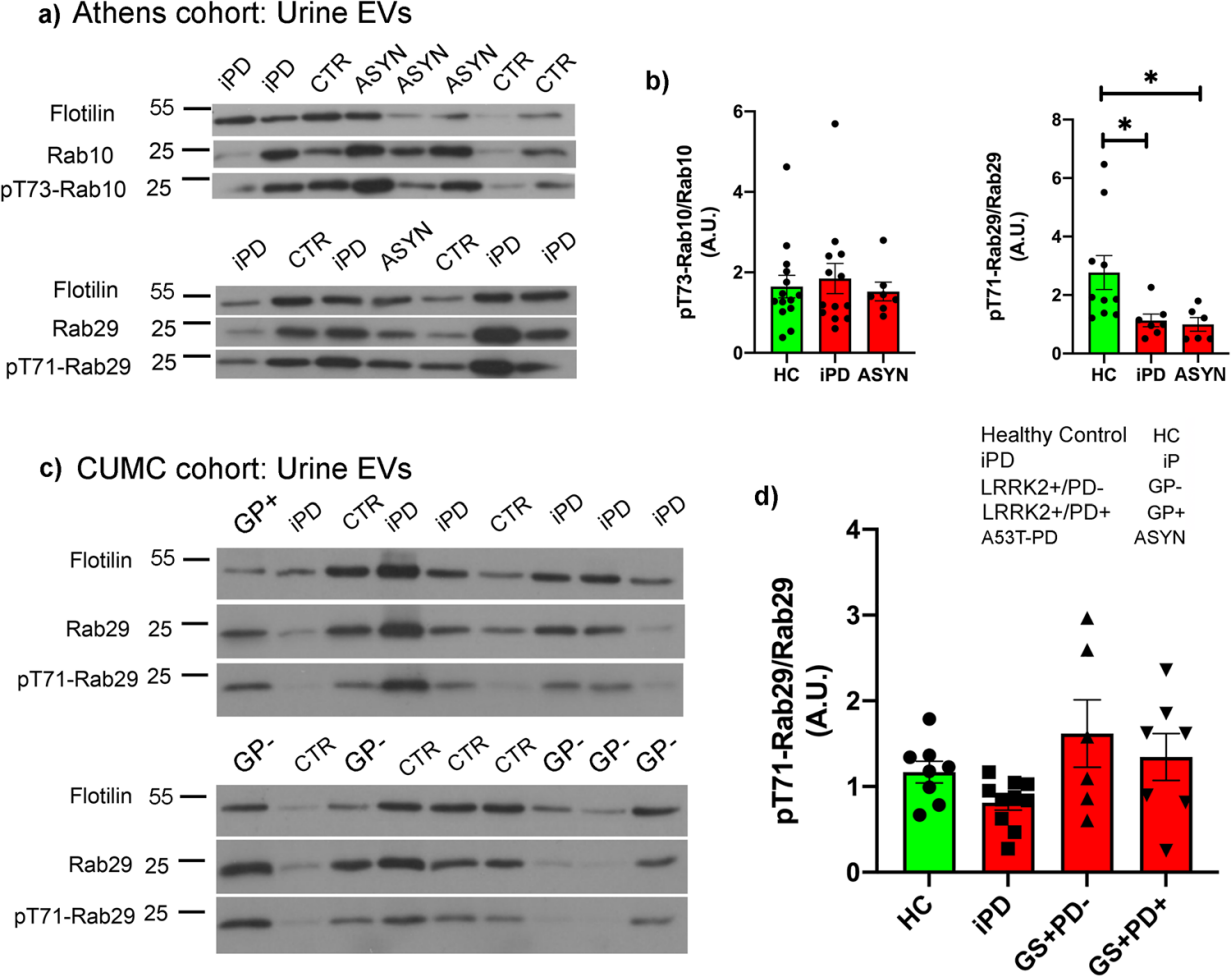
Phosphorylation of Rab10 is unaffected in urinary EVs but phosphorylation of Rab29 is decreased in two PD cohorts. EVs from urine were purified by ultracentrifugation, and extracts separated by SDS-PAGE. The membranes were probed with total or phosphorylated Rab10 and 29, and flotilin as a marker for EVs. Band intensities of phosphorylated Rab10 or Rab29 were normalized to total Rab. **a**, **c** Representative blots showing phosphorylated Rab10 and Rab29; and quantified in (**c**, **d**). Error bars in all data plots represent SEM. ANOVA was performed to identify significant differences between groups, with Tukey’s post-hoc tests. *p < 0.05.

In the two G2019S-*LRRK2* carrier groups, we found a divergence in Rab10 phosphorylation that was strictly linked to disease status. Affected carriers of this mutation exhibited a significant increase in normalized pT73-Rab10 levels, similar to iPD patients, compared to healthy controls as well as G2019S+ PD−; whereas pRab10 levels were not different from control subjects in non-manifesting G2019S-*LRRK2* carriers (Fig. 1a, b). We have previously shown from the same cohort^6^, that the in vitro kinase activity of LRRK2 purified from PBMCs of both G2019S carrier groups is significantly increased compared to healthy control and iPD patients. Thus, in a cellular milieu, the disease context is clearly impacting the phosphorylation of LRRK2 substrates, even in the presence of a hyperactive enzyme. In these cohorts however, we did not observe significant correlations between phosphorylated Rab10 levels and multiple clinical outcomes (i.e. UPDRS-III, MoCA, disease duration). Finally, in PBMCs from affected carriers of the A53T mutation in α-synuclein (ASYN), phosphorylation of Rab10 was unchanged in PBMCs compared to healthy controls (Fig. 2a, b).

In addition to Rab10 phosphorylation, we assessed the induction of phosphorylation of another LRRK2 substrate, Rab29, in PBMCs from each group. We could readily detect robust expression of Rab29 in PBMCs from each group, and this did not significantly differ between each group. Interestingly, in most samples, we could not detect phosphorylated Rab29 (T71), even in the presence of a signal boost reagent (SignalBoost Immunoreaction, Sigma; not shown), suggesting that within PBMCs, Rab29 is not a particularly prominent LRRK2 substrate, either in the healthy control population or in any of the PD cohorts; or that specific Rab29 phosphatases are particularly active in these cells.

### Changes in Rab10 and Rab29 phosphorylation in urinary extracellular vesicles/exosomes

In extracts of urinary extracellular vesicles (EVs), the detection of LRRK2 was highly variable; in many samples, total and pS1292-LRRK2 was un-detectable (Supplementary Fig. 2b, c), even in the presence of the SignalBoost detection reagent. However, we were able to easily detect both total and pT73-Rab10 in extracts of EVs by Western immunoblotting (Fig. 2a). The pattern of Rab10 phosphorylation differed in urinary EVs; where, in contrast to PBMCs, phosphorylated Rab10, normalized to total Rab10, in EVs from iPD and A53T-ASYN patients were unchanged compared to healthy controls (Fig. 2b). Interestingly, whereas we could not detect phosphorylated Rab29 in extracts of PBMCs from this cohort, we were able to detect pT71-Rab29 in urinary EVs; and found a significant decrease in pRab29 (normalized to total Rab29) in EVs from A53T-PD patients compared to healthy controls (Fig. 2c). Phosphorylated Rab29 levels in urinary EVs from iPD patients were also reduced compared to healthy controls (Fig. 2c); however the drop in pRab29 levels did not correlate with any of the clinical outcomes reported (e.g. UPDRS-III, MoCA, or disease duration). We did observe a slight decrease in pRab29 in urine EVs of iPD patients from the CUMC cohort, similar to the Athens cohort, which only attained statistical significance following a two-tailed t-test (not shown), and not following the ANOVA performed on the full data set. Interestingly, we did not observe a correlation between levels of pRab10 in PBMCs and urine EVs, in either cohort. The reason for this is likely due to the different cellular sources of the Rab10 in each sample. There are multiple cell types in the PBMC population that express detectable levels of Rab10^10^, and similarly it is not possible, in the preparations we employed, to determine the cellular source of the EVs recovered from the patient urine samples. A more targeted study of specific blood cell types and immuno-isolated EVs (e.g. from urine, plasma, or even CSF) would be better suited to reveal such a correlation.

In addition to PBMCs and urine, some study participants from the Athens cohort were asked to provide an additional sample from which we purified CD14+ monocytes. A separate manuscript describing in more detail these samples, focusing on changes in LRRK2 levels and activity, is being prepared; but in the current study we also assessed changes in Rab10 phosphorylation. In neither the iPD study group, nor the A53T-ASYN PD group, were we able to detect significant changes in phosphorylation of Rab10 compared to healthy controls. These cells express high levels of LRRK2^10^; however its phosphorylation of Rab10 at Thr73 appears unchanged in either PD study group, at least among the small sample size assessed here (Supplementary Fig. 3). We were not able to assess Rab10 phosphorylation in carriers of the G2019S mutation in the LRRK2 gene; where we might expect to detect elevated phosphorylation within the affected group, as we have in PBMCs from this cohort.

## Discussion

In the present study, we assessed Rab GTPase phosphorylation in PBMCs and urinary exosomes from multiple PD cohorts collected at different clinical centers. We recently reported several LRRK2-based outcome measures in a cohort of G2019S-*LRRK2* carriers (both healthy and affected) from the CUMC-Movement Disorders center. In PBMCs from these individuals, we found a significant elevation in phosphorylation of the LRRKtide peptide substrate in both G2019S carrier groups, compared to healthy controls and idiopathic PD patients^6^. This is not surprising given the conserved motif that this mutation lies within in the LRRK2 kinase domain (e.g. ref. ^14^). In the same report, we could not detect changes in the in vitro kinase activity of LRRK2 isolated from iPD PBMCs^6^.

In PBMCs from G2019S carriers, we report here increased phosphorylation of Rab10 (T73) *only* in affected carriers of this mutation, healthy G2019S carriers exhibited pT73-Rab10 levels similar to healthy controls. Thus, the isolated enzyme bearing the Gly to Ser substitution in the critical *DYG* kinase domain motif exhibits elevated intrinsic kinase activity, regardless of disease status^6^; however, in a cellular context the disease status clearly interacts with the genotype to modulate Rab10 phosphorylation, at least in peripheral immune cells. In other words, in vitro kinase activity is a good predictor of LRRK2 mutation status, but not of disease status. In another recent study, combining western immunoblotting and targeted mass-spectrometry measures of phosphorylated Rab10, it was reported that T73-Rab10 levels in a small sample of carriers of the R1441G were significantly elevated in neutrophils compared to healthy control subjects^12^. Unlike the present study, phosphorylated Rab10 levels from carriers of the G2019S-*LRRK2* mutation were not different from healthy controls, regardless of disease status^12^; however in an earlier study also using an MS-based approach, pT73-Rab10 levels in neutrophils were approximately 2-fold higher in a set of G2019S carriers^15^. We did not detect changes in Rab10 phosphorylation in CD14+ monocytes in either the iPD or the A53T-ASYN patient groups, suggesting that the cellular source of increased pRab10 in the iPD and G2019S+PD+ PBMCs is not this subset of monocytes, but perhaps neutrophils (e.g.^12^), or other monocyte subsets, such as CD16+ monocytes which have been shown to exhibit increased LRRK2 expression in PD^16^. Additionally, using a novel phospho-specific Rab10 antibody in an ELISA-based outcome measure, Wang and colleagues reported slightly elevated pRab10, normalized to LRRK2 levels, in PBMCs of both healthy and affected G2019S carriers^8^. It should be noted that in this study, the authors reported a slight but significant decrease in LRRK2 expression in both G2019S-LRRK2 groups, which may account for the elevated pRab10 levels when normalized to LRRK2 instead of total Rab10. Nevertheless, while the current study remains semi-quantitative in its approach, the study by Wang and colleagues represents an important advance in the field in terms of more quantitative assessments of changes in LRRK2 pathway signaling in PD.

In PBMCs from iPD or A53T-PD patients, we do not detect changes in the intrinsic kinase activity of isolated LRRK2 (ref. ^6^; and manuscript in preparation); whereas in two independent cohorts (CUMC and Athens), we find increased phosphorylation of Rab10 in PBMCs from iPD patients. Not surprisingly, there is considerable variation in phosphorylated Rab10 levels in these clinical samples, which is reflective of course of LRRK2 kinase activity but also potentially other kinases, as well as the activity of PPM1H, a phosphatase implicated in Rab10 de-phosphorylation^17^. Further, deeper analyses should be performed to determine if specific subsets of patients exhibit increased Rab10 phosphorylation, which would likely be correlated with other biochemical outcomes or other specific clinical measures. In this study, however, we did not observe any correlations with clinical outcomes (e.g. UPDRS-III and others). Previous reports have not revealed differences in Rab10 phosphorylation in this patient group (e.g. ref. ^11^); the reasons for this discrepancy are unclear, however may be related to the normalization methods taken in each study. Rab10 phosphorylation is reported to be increased in post-mortem brain from iPD patients^5^; thus, taken together with our present findings, it suggests that increased phosphorylation of this key substrate, and by extension LRRK2 kinase activity, is a systemic phenomenon in this disease cohort. The lack of significant increases in Rab10 phosphorylation in A53T-PD patients suggests that there are multiple PD progression pathways, depending on the form of the disease. Alternatively, in this specific patient group, with an earlier age of disease onset (see Table 1), it is possible that a transient increase in Rab10 phosphorylation occurred at an earlier point in the disease course. And finally, given that the sample number was significantly less in this patient group, it is also possible that with additional samples subtle changes in Rab10 phosphorylation in PBMCs would be revealed. It is not known at this point if there are detectable changes in Rab10 phosphorylation in brains of A53T-PD patients; however we are currently assessing neuronally derived exosomes for changes in LRRK2 and Rab phosphorylation. Additionally, a longitudinal study with sufficiently large patient groups assessing multiple markers is critically needed to address these questions.

**Table 1:** Summary of demographics of study participants (Athens cohort).

	Healthy controls (HC)	Idiopathic PD (iPD)	A53T-*SNCA* PD	*p* value	Statistical tests
Gender (M:F)	7:9	14:6	4:4		
Age	55 (36–75)	72 (47–85)	51 (30–73)		
Age at onset	na	66 (42–80)	46 (27–68)	*p* = 0.0011	MWU (iPD vs A53T-PD)
LEDD	na	629 (0–1865)	758 (150–1500)	ns	MWU (iPD vs A53T-PD)
UPDRS-III	0	30 (0–91)	31 (9–53)	ns	MWU (iPD vs A53T-PD)
MoCA	26 (17–30)	19 (4–26)	26 (16–30)	HC vs. iPD (*p* = 0.0025) HC vs. A53T (ns)	MWU
H&Y	0	2 (0–4)	2 (1–3)	ns	MWU (iPD vs A53T-PD)

*H&Y* Hoehn & Yahr PD scale, *MoCA* Montreal Cognitive Assessment, *LEDD* levo dopa equivalent daily dosage, *UPDRS-III* united PD rating scale (part 3).

A second key finding we report here is the loss of phosphorylation of Rab29 (Thr71) in urinary exosomes from iPD and A53T-PD patients. Recently, it was reported by two independent groups that over-expression of Rab29 can trigger the recruitment of LRRK2 to intracellular membranes and the trans-Golgi network (TGN) leading to its activation^18,19^; suggesting a regulatory cycle between LRRK2 activity and Rab29. In our clinical biofluid samples, the loss of phosphorylation of Rab29 in urine exosomes from iPD and A53T-PD patients, releasing its inhibition, would be consistent with increased activation of LRRK2 (e.g. increased pT73-Rab10 in the iPD group). While we could not detect changes in phosphorylation of Rab10 in this cohort, the reduction in Rab29 phosphorylation is the first indication of possible changes in LRRK2 activation in clinical samples from PD patients carrying the A53T-*SNCA* mutation; and bolsters the notion that LRRK2 function plays an important role in PD in general. A summary of our findings in the current study, in the context of our previously reported findings from one of the two cohorts (from CUMC) is presented in Table 2. Our findings reinforce the need to expand our pallet of validated LRRK2-pathway outcome measures to include additional targets as well as multiple biofluid types^20^.

**Table 2:** Summary of demographics of study participants (CUMC cohort).

	Healthy controls (HC)	Idiopathic PD (iPD)	LRRK2+/PD−	LRRK2+/PD+	*p* value	Comparisons
Sex (M:F)	8:12	18:12	4:5	4:5	ns	
Age	68.3 (57–85)	67.5 (45–86)	57.1 (38–69)	74 (64–90)	*p* < 0.01–0.05	GS+/PD− vs. all groups
Age at onset	na	60.5 (27–84)	na	64.6 (54–83)	ns	
LEDD	na	466.3 (0–1520)	na	631.3 (300–1625)	ns	
UPDRS-III	0	18.9 (5–46)	0	20.6 (12–28)	ns	
MoCA	27.8 (24–30)	26.4 (15–30)	28.4 (27–30)	26.5 (22–29)	*p* < 0.05	iPD vs. GS+/PD−

*H&Y* Hoehn & Yahr PD scale, *MoCA* Montreal Cognitive Assessment, *LEDD* levo dopa equivalent daily dosage, *UPDRS-III* united PD rating scale (part 3).

## Methods

### Study participants

The samples that we assessed were collected as part of two separate biomarker studies. The demographics of a subset of the clinical cohort (healthy controls, iPD, G2019S*-LRRK2*/PD+ & G2019S-*LRRK2*/PD−) followed at Columbia University Medical Center (New York, USA) have been reported in detail in a separate manuscript^6^, and are summarized in Table 1. The second cohort (healthy controls, iPD, & A53T-*SNCA* PD) was derived from patients followed at the Departments of Neurology at Henry Dunant Hospital Center (Athens, Greece) and Aiginiteio University Hospital (Athens, Greece). All samples were collected, processed and coded at the time of collection so that the analyses of the samples were performed by researchers blinded to the PD and mutation status. The study was approved by the institutional ethical review boards of both Columbia University (RNA) as well as Biomedical Research Foundation of the Academy of Athens (BRFAA; HJR), and all participants signed written informed consent. Thus, in this study we have included two independent groups of healthy controls and iPD patients. Demographics and key clinical features of the Athens cohort are summarized in Table 3. The samples from each cohort were analyzed independently. In some cases, fewer samples were included in a specific analysis than were originally collected due to technical issues with detection of the specific target in the sample matrix. For example, in the samples where signal from the total protein (for normalization) was missing, these were excluded from the analyses.

**Table 3:** Summary of LRRK2-specific changes in two PD cohorts.

	Total LRRK2	In vitro kinase activity	pS935-LRRK2	pS1292-LRRK2 (WB)	pT73-Rab10 (WB)	pT71-Rab29 (WB)
Healthy Control (HC)	Baseline	Baseline	Baseline	Detectable only	Baseline	Baseline
Idiopathic PD (iPD)	Like HC	Like HC	Elevated (2 cohorts; Melachroinou et al., 2020; manuscript in prep.)	Detectable only	Elevated (2 cohorts)	Decreased (Ur-EVs)
G2019S+ PD+	Like HC	Elevated (Melachroinou et al., 2020)	Slight decrease (Melachroinou et al., 2020)	Detectable only	Elevated	ND
G2019S+ PD−	Like HC	Elevated (Melachroinou et al., 2020)	Slight decrease (Melachroinou et al., 2020)	Detectable only	Like HC	ND
A53T-*SNCA* PD	Manuscript in prep.	Manuscript in prep.	Manuscript in prep.	Detectable only	Like HC	Decreased (Ur-EVs)

### PBMC & urinary exosome isolation

PBMCs were isolated from whole blood collected in Heparin-coated Vacutainer collection tubes (BD), using protocols established in our recent work^6^. Some aliquots were divided into two samples and treated with DMSO or the LRRK2 inhibitor MLi-2, then collected/washed in PBS and snap frozen as a cell pellet in dry ice, and stored at −80 °C. Urine (~50–200 ml) was collected from participants in the morning of their clinic visit and tested for urinary infections.

Exosomes were isolated as described from the group of West and colleagues^3^, and lysed under the same conditions as PBMCs. Briefly, an initial centrifugation took place for 30 min, at 4500 g, at 4 °C for debris clearance and the supernatant was stored at −80 °C in 50 ml falcon tubes. After quickly thawing the urine in a 42 °C waterbath, the EVs were isolated by ultracentrifugation (Sorval Discovery, 100 SE) at 100,000 g, for 1 h at 4 °C. EV pellets were lysed with 50 μl lysis buffer (50 mM Tris-HCl, pH 7.5, 1 mM EGTA, 270 mM Sucrose, 1% Triton + 1X protease/phosphatase inhibitors added fresh), for 1 h on ice. Lysates were then centrifuged at 13,000 rpm, for 15 min at 4 °C and the supernatant was collected and stored at −80 °C. Parallel EV pellets from sample preps were resuspended in PBS after ultracentrifugation for TEM imaging (shown in Supplementary Fig. 2d). For the preparation of urinary exosomes for visualization by transmission electron microscopy (TEM), the exosome pellets were resuspended in PBS, and 2 μl of exosome suspension removed for imaging. Apart from the TEM confirmation of EV size and shape, and positive immunoreactivity for the exosomal marker flotillin, we confirmed that extracts of urine EVs were also positive for the exosomal markers TSG101 (Supplementary Fig. 2e).

Where possible, in the Athens cohort, we collected CD14+ monocytes in addition to PBMCs and urine. Blood was collected in K_2_-EDTA Vacutainer collection tubes (BD) and 15 ml was transferred to a 50 ml falcon and 750 μl ‘’Isolation Cocktail” from the EasySep^TM^ Direct Human Monocyte Isolation Kit (StemCell Technologies) was added. ‘’RapidSphere Magnetic Beads” from kit were vortexed for 30 s before use and added to the blood sample. Blood was mixed gently by tube inversion and incubated for 5 min, at RT. The volume was increased to 50 ml with 1 mM EDTA-PBS Ca2^+^/Mg2^+^ free (Gibco), and the solution was gently mixed. The tube was placed into a magnet (StemCell Technologies) without the lid, for 10 min, to immobilize the magnetic beads along the wall of the centrifuge tube. The enriched monocyte cell suspension was carefully removed (leaving ~10 ml behind) into a new 50 ml falcon, where 750 μl of fresh magnetic beads were added and tube was gently mixed followed by 5 min RT incubation. The cell suspension was again removed (leaving ~5 ml behind) and to ensure complete removal of magnetic beads, the tube was placed in the magnet for another 10 min, at RT. Purified CD14^+^ monocytes were then transferred to a new falcon (leaving ~5 ml behind) and the 30 ml suspension was increased with 1 mM EDTA-PBS Ca2^+^/Mg2^+^ to a final volume of 40 ml. Centrifugation for 5 min, at 335 g, at RT followed and the cell pellet was washed once with 1X PBS and stored at −80 °C. Prior to biochemical analysis, pellets were lysed with 50 μl lysis buffer (50 mM Tris-HCl, pH 7.5, 1 mM EGTA, 270 mM Sucrose, 1% Triton + 1X protease EDTA-free/phosphatase cocktail inhibitors (Roche) added fresh), for 30 min on ice. Lysates were then centrifuged at 45,000 rpm, for 1 h, at 4 ^o^C and S/N was collected and stored at −80 °C.

For western immunoblotting, protein samples were separated by SDS-PAGE, and the membranes blocked in either 5% Milk/TBST (for Rab10, Rab29, GAPDH, LRRK2, and Flotilin-1) or in 5% BSA/TBST (for pS1292-LRRK2, pT73-Rab10 and pT71-Rab29), for 1 h at RT. The primary antibodies used were as follows: Rab10 (ab237703 Abcam, rabbit, 1:1000), Rab29 (ab199644 Abcam, rabbit, 1:1000), Flotilin-1 (sc-133153 Santa Cruz, mouse, 1:1000), LRRK2 (ab133474 Abcam rabbit, clone c41–2, 1:1000), pT73-Rab10 (ab230261 Abcam, rabbit, 1:1000) and pT71 Rab29 (ab241062 Abcam, rabbit, 1:1000), and pS1292-LRRK2 (ab203181 Abcam, clone MJF-19–7–8, 1:1000), shaking overnight at 4 °C. Initially, full-length membranes were probed with a primary antibody (as indicated, representative blot is shown in Supplementary Fig. 1a), and then stripped before re-probing. In subsequent immunoblots, the membranes were cut horizontally around the molecular weight of the specific protein, to preserve the limited volume of the clinical samples, and probe each membrane with multiple antibodies. In some cases, where the membranes were probed with anti pS1292-LRRK2 antibodies, we included Signal Boost Immunoreaction Reagent (Sigma) in the antibody diluent for both the primary and secondary antibody incubation steps. Membranes were then washed 3x for 10 min in 1X TBST and incubated with HRP-conjugated secondary antibodies (Millipore AP132P anti-rabbit or Millipore AP124P anti-mouse, 1:5000), for 1 h at RT. Three more washes followed and membranes were exposed to ECL and developed using an automatic film developer. Films were scanned and quantified using ImageJ, with the final Figures compiled using Adobe Photoshop.

For quantification and normalization of the phosphorylated protein, the ratio of band intensity of phosphorylated:total protein was determined. Because of the semi-quantitative nature of western immunoblotting, band intensities were measured from films taken from similar exposures wherever possible. The samples were run in a blinded fashion (i.e. coded), and band intensities were obtained by an independent researcher, before the sample ID’s were decoded. Band intensities of phosphorylated Rab10 were first normalized to total Rab10 expression; and, alternatively to total levels of LRRK2 determined by ELISA^6^.

### Statistical analyses

For statistical comparison of the different study groups, we employed ANOVA with Tukey’s post-hoc multiple comparisons, or if required non-parametric Kruskal–Wallis tests & Dunn’s post-hoc test; or two-tailed *t*-tests where indicated.

## Supplementary information

Supplementary Material

Supplementary Figure 1

Supplementary Figure 2

Supplementary Figure 3

## Data Availability

Non-identifying data generated during this study can be made available from the corresponding author upon reasonable request.
