# Supplementary material for: Distinct profiles of LRRK2 activation and Rab GTPase phosphorylation in clinical samples from different PD cohorts
Source: NPJ Parkinsons Dis. 2022 Jun 8;8:73. doi: 10.1038/s41531-022-00336-5 (PMC9177829; doi:10.1038/s41531-022-00336-5)
Supplement: Supplementary file 1 — Supplementary Material [file 41531_2022_336_MOESM1_ESM.docx]

**Supplementary Material**

**Results**.

*Phosphorylated S1292-LRRK2 is barely detectable in peripheral blood cells of PD patients*. Prior to assessments of Rab phosphorylation in clinical samples as an index of LRRK2 kinase activity, auto-phosphorylation of LRRK2 itself, at Ser1292, was used to reflect kinase activity. Increased phosphorylation at this residue has been reported in LRRK2 present in urinary exosomes from both idiopathic as well as G2019S-LRRK2 PD patients ^1,2^. In PBMCs, however, while total LRRK2 expression is readily detectable, phosphorylation at this residue is very weak, unless a signal boost reagent was included. We loaded increasing amounts of PBMC protein extract and probed the membranes for pS1292 and total LRRK2. The inclusion of the Signalboost reagent may alter the linearity of the detection of pS1292-LRRK2 by western immunoblot (Supplementary Fig. 2b) in unknown ways; thus we elected not to use this pharmacodynamics readout in assessing LRRK2 function in PBMCs.

*pS1292-LRRK2 in urine exosomes*. Previous reports have assessed auto-phosphorylation of LRRK2 at the Ser1292 site, and found increased levels in urinary exosomes from iPD and G2019S-PD patients ^1,2^. We assessed pS1292-LRRK2 by Western immunoblotting in extracts of urine exosomes. Detection of LRRK2, as well as pS1292-LRRK2, was quite variable in samples across the subject groups, with LRRK2 being un-detectable in many cases (Supplementary Figure 2c). A representative TEM image is shown in Supplementary Figure 2d; depicting the size and shape of exosomes recovered from urine by ultracentrifugation.

References.

1 Fraser, K. B., Moehle, M. S., Alcalay, R. N., West, A. B. & Consortium, L. C. Urinary LRRK2 phosphorylation predicts parkinsonian phenotypes in G2019S LRRK2 carriers. *Neurology* **86**, 994-999, doi:10.1212/WNL.0000000000002436 (2016).

2 Fraser, K. B. *et al.* Ser(P)-1292 LRRK2 in urinary exosomes is elevated in idiopathic Parkinson's disease. *Mov Disord*, doi:10.1002/mds.26686 (2016).
