# Supplementary figures and images for: Distinct profiles of LRRK2 activation and Rab GTPase phosphorylation in clinical samples from different PD cohorts
Source: NPJ Parkinsons Dis. 2022 Jun 8;8:73. doi: 10.1038/s41531-022-00336-5 (PMC9177829; doi:10.1038/s41531-022-00336-5)

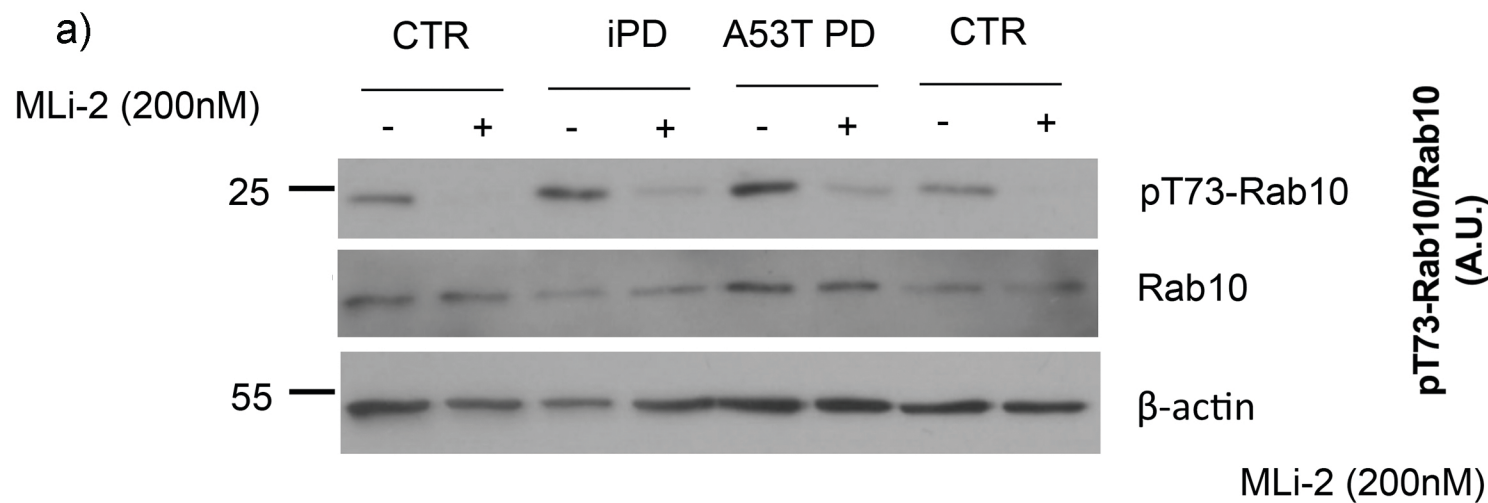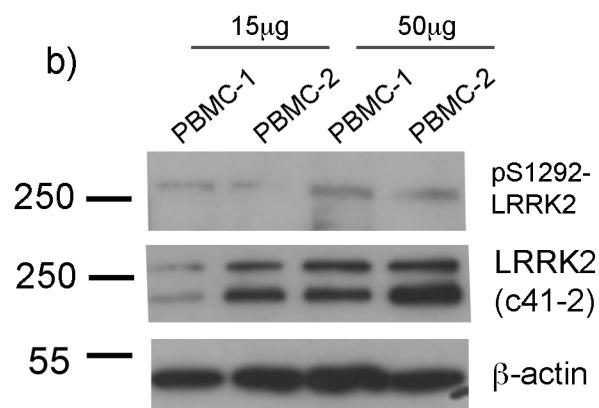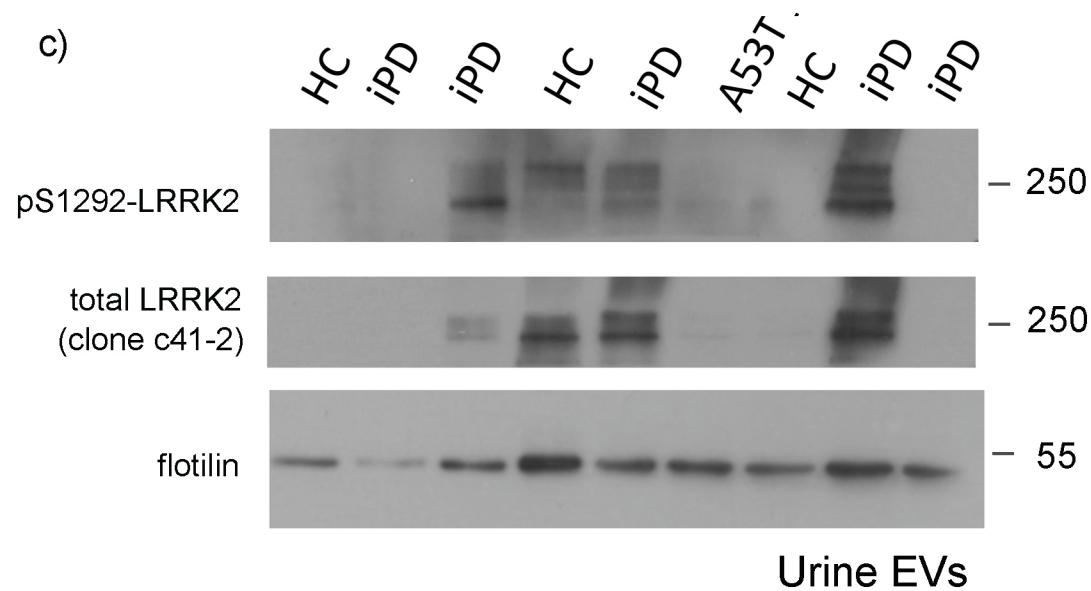

**d) Urine EVs**

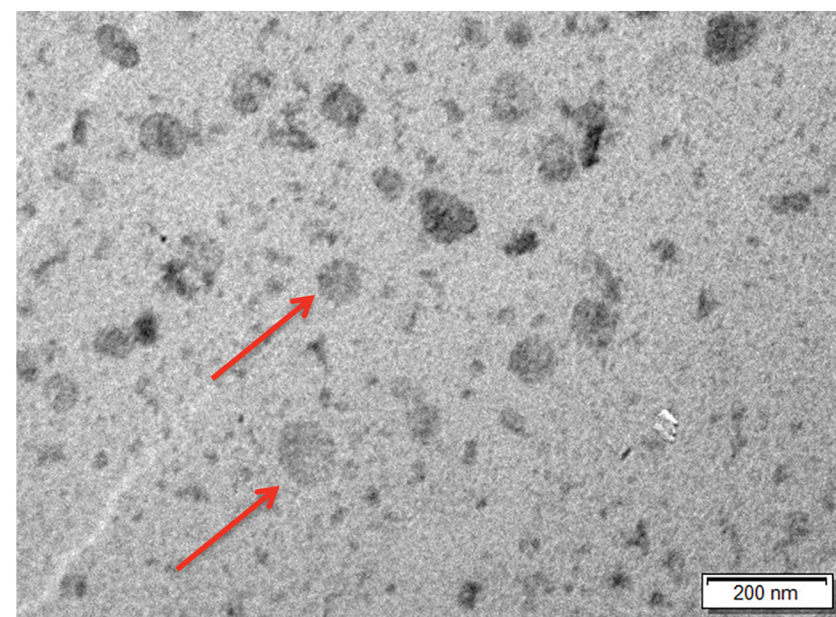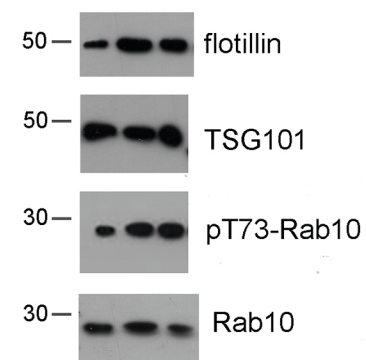

Supplement: Supplementary file 3 — Supplementary Figure 2 [file 41531_2022_336_MOESM3_ESM.pdf]

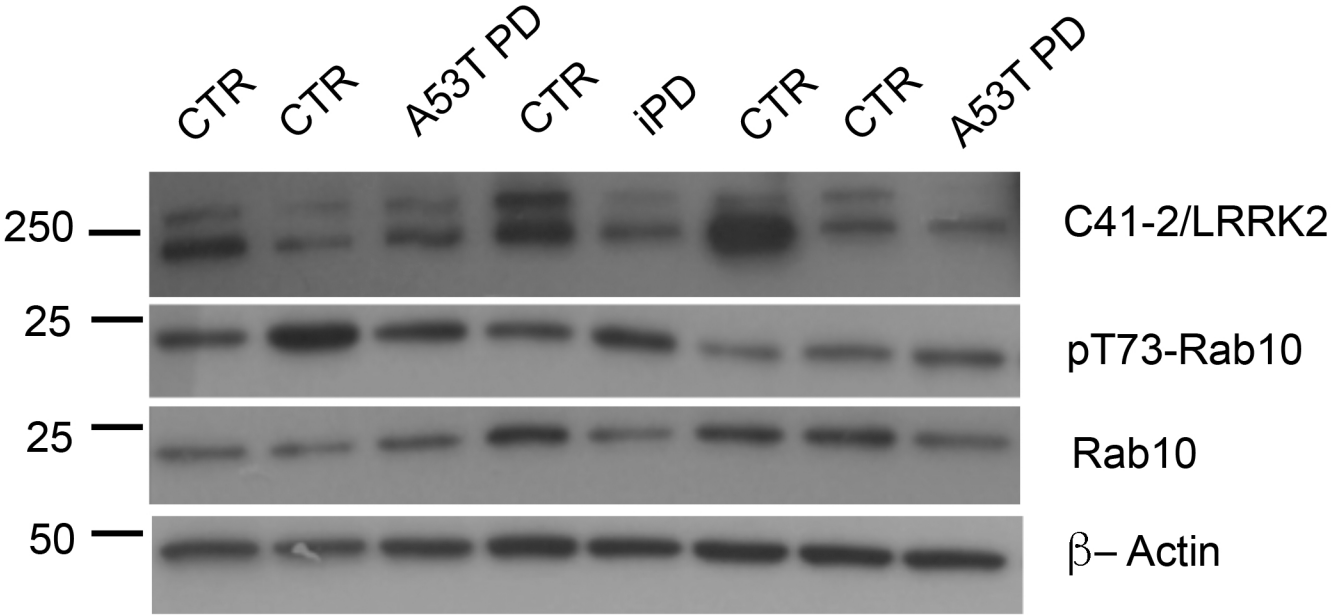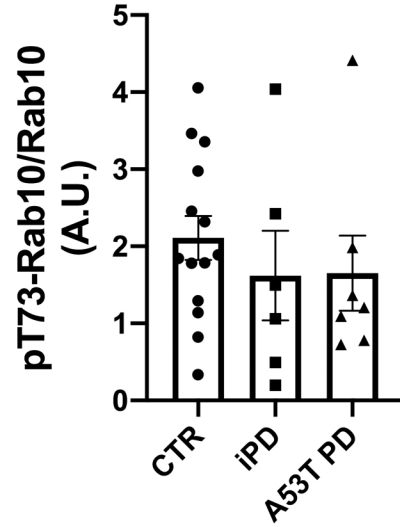

Supplement: Supplementary file 4 — Supplementary Figure 3 [file 41531_2022_336_MOESM4_ESM.pdf]
