# Supplementary material for: Distinct profiles of LRRK2 activation and Rab GTPase phosphorylation in clinical samples from different PD cohorts
Source: NPJ Parkinsons Dis. 2022 Jun 8;8:73. doi: 10.1038/s41531-022-00336-5 (PMC9177829; doi:10.1038/s41531-022-00336-5)
Supplement: Supplementary file 2 — Supplementary Figure 1 [file 41531_2022_336_MOESM2_ESM.pdf]

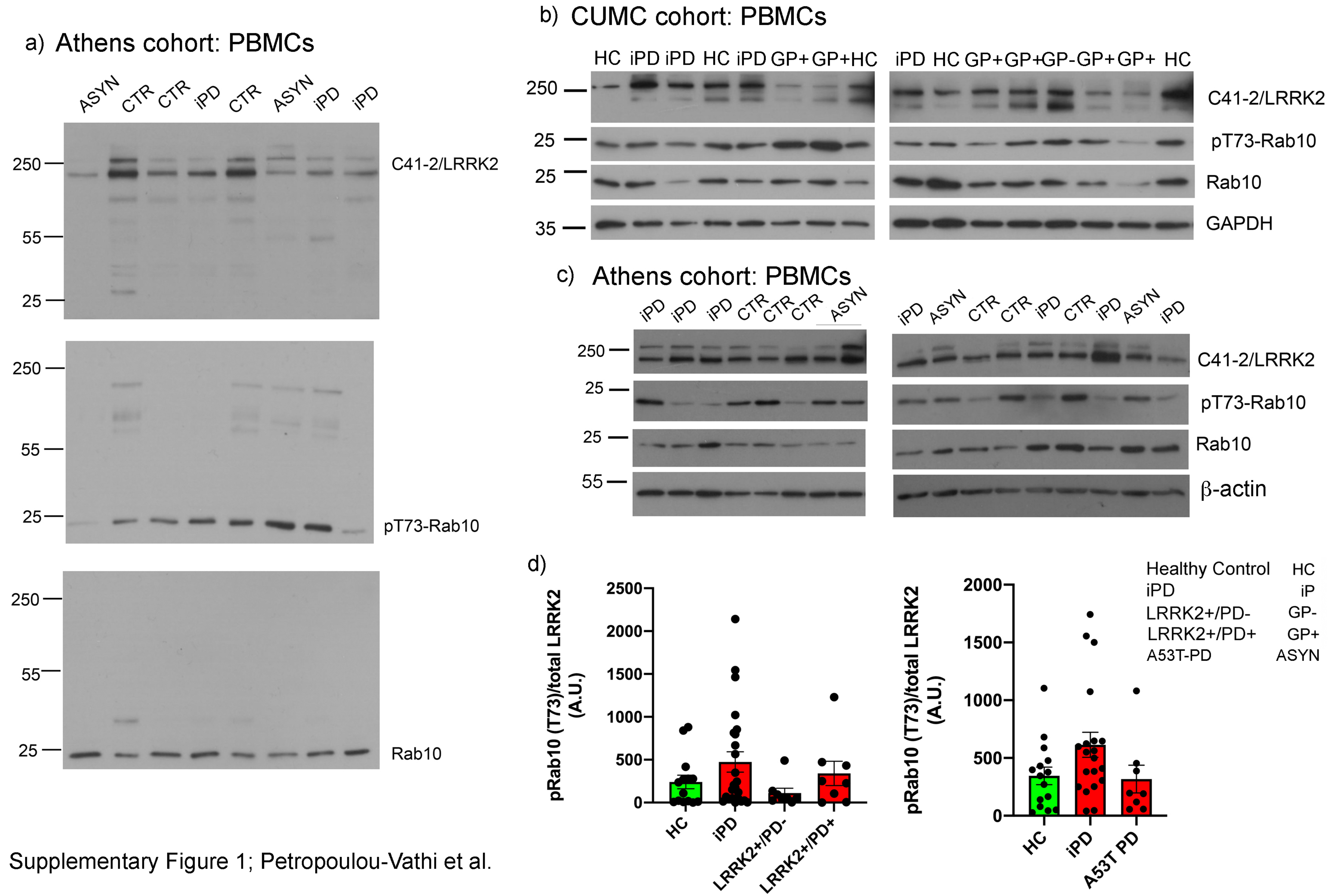


Supplementary Figure 1; Petropoulou-Vathi et al.
